# Fecal calprotectin levels in acute anterior uveitis in patients with spondyloarthritis

**DOI:** 10.5935/0004-2749.20230008

**Published:** 2023

**Authors:** Caio Cézar Gazim, Angelo Antonio de Borba, Grace Kelly Pelicioni de Castro, Juliana Simioni, Marcelo Luiz Gehlen, Odery Ramos Júnior, Thelma Larocca Skare

**Affiliations:** 1 Ophthalmology Unit, Hospital Universitário Evangélico Mackenzie, Curitiba, PR, Brazil; 2 Ophthalmology Unit, Hospital de Olhos do Paraná, Curitiba, PR, Brazil; 3 Rheumatology Unit, Faculdade Evangélica Mackenzie do Paraná, Curitiba, PR, Brazil; 4 Ophthalmology Unit, Faculdade Evangélica Mackenzie do Paraná, Curitiba, PR, Brazil; 5 Gastroenterology Unit, Faculdade Evangélica Mackenzie do Paraná, Curitiba, PR, Brazil

**Keywords:** Calprotectin, Uveitis, Spondyloarthritis, Inflammatory bowel diseases, Biomarkers, Calprotectina, Uveíte, Espondiloartropatias, Doenças inflamatórias intestinais, Biomarcadores

## Abstract

**Purpose:**

This study measured fecal calprotectin levels in a series of patients with anterior uveitis in order to determine whether anterior uveitis patients with associated spondyloarthritis have higher levels of fecal calprotectin than patients with anterior uveitis of other etiologies. A third group of patients with spondyloarthritis without uveitis was also evaluated to understand the role of acute anterior uveitis in increasing fecal calprotectin.

**Methods:**

In this cross-sectional study, 28 patients were divided into three groups: (a) Group 1, spondyloarthritis and uveitis (n=9); (b) Group 2, spondyloarthritis without uveitis (n=10); and (c) Group 3, uveitis without spondyloarthritis (n=9). The levels of fecal calprotectin were determined.

**Results:**

Groups 1 and 2 showed higher median fecal calprotectin levels (101.0 and 93.0 µg/g, respectively) compared with Group 3 (9.0 µg/g) (p=0.02). However, no relationship between fecal calprotectin levels and the presence of uveitis with spondyloarthritis could be demonstrated.

**Conclusion:**

Patients with spondyloarthritis with or without acute anterior uveitis have significantly elevated levels of fecal calprotectin. This test may be useful for differentiating spondyloarthrit-associated uveitis from uveitis of other etiologies.

## Abstract (pt)

**Objetivo:**

Este estudo avaliou os níveis de calprotectina fecal em uma série de pacientes com uveíte anterior na tentativa de determinar se pacientes com uveíte associada com espondiloartrites apresentam níveis mais elevados desta proteína do que pacientes com uveíte anterior de outras etiologias. Um terceiro grupo com espondiloartrites sem uveíte também foi incluído na avaliação para entendimento do papel da uveíte anterior no aumento da calprotectina fecal.

**Métodos:**

Estudo transversal de 28 pacientes divididos em três grupos: (a) com espondiloartrites e uveíte (n=9); (b) com espondiloartrites sem uveíte (n=10) e (c) com uveíte sem espondiloartrites (n=9). A dosagem de calprotectina fecal foi avaliada.

**Resultados:**

Pacientes com uveíte anterior associada a espondiloartrites apresentaram valores medianos maiores de calprotectina fecal (101 µg/g) que os valores dos pacientes com uveíte sem espondiloartrites (9 µg/g), pacientes com espondiloartrites sem uveíte que também demonstraram valores maiores (93.0 µg/g) que os dos pacientes com uveíte sem espondiloartrites (p=0,02).

**Conclusão:**

Pacientes com espondiloartrites com e sem uveíte anterior aguda demonstraram níveis significativamente elevados de calprotectina fecal. Este teste pode ser útil na diferenciação entre uveítes associadas com espondiloartrites de uveítes de outras etiologias. Entretanto, não foi possível demonstrar associação entre o aumento dos níveis de calprotectina fecal e a presença da uveíte em espondiloartrites.

## INTRODUCTION

Spondyloarthritis (SpA) encompasses a group of rheumatic diseases with common clinical, laboratory, and image findings. Some of the diseases are ankylosing spondylitis, reactive arthritis, psoriatic arthritis, and arthritis associated with inflammatory bowel diseases, such as Crohn’s or ulcerative colitis^(1,2)^. SpA also shares an association with inflammatory eye diseases, such as uveitis, a condition that usually affects the anterior segment of the eye^(1)^.

Anterior uveitis may appear with other rheumatic diseases, such as idiopathic juvenile arthritis and Behçet’s disease, complicating the differential diagnosis^(3)^. In addition, uveitis may be found in patients with previously undiagnosed SpA, especially in those with few clinical findings and scarce radiological signs, making the diagnosis even more difficult^(4)^.

Calprotectin, also known as S100A8/S100A9, is a heterodimer formed by two intracellular proteins linked to calcium and has antimicrobial properties^(5,6)^. This complex is released by monocytes and granulocytes in the area of inflammation. Fecal calprotectin levels are considered an excellent biomarker of inflammatory intestinal activity^(7)^.

There is an association between serum calprotectin levels and uveitis activity^(8)^. Wang et al. showed that calprotectin is elevated in uveitis patients compared with non-uveitic controls and that patients with ankylosing spondylitis and uveitis present significantly higher serum calprotectin levels compared with patients with acute anterior uveitis without ankylosing spondylitis^(8)^. Therefore, serum calprotectin levels could be used to determine intraocular inflammation^(8)^.

SpA patients may have subclinical intestinal inflammation. Alterations in the intestinal microbiome (also known as dysbiosis) are implicated in the occurrence of rheumatic diseases and their inflammatory flares^(7)^, which may be associated with the breakdown of intestinal wall integrity, causing increased levels of fecal calprotectin^(7,9)^. Dysbiosis may also be found in uveitis, but the role of fecal calprotectin in this context has not been studied^(10)^.

This study measured fecal calprotectin levels in a series of patients with anterior uveitis in order to determine whether uveitis patients with associated SpA have higher levels of this biomarker than patients with anterior uveitis of other etiologies. A third group of patients with SpA without uveitis was also evaluated in order to identify uveitis as an isolated factor in increasing fecal calprotectin levels.

## METHODS

### Study Subjects and Design

This study was approved by the Committee of Ethics in Research of the Mackenzie Evangelic Hospital of Paraná. In this small-sample, cross-sectional study, 28 patients were divided into three groups: Group 1, SpA with acute uveitis (n=9); Group 2, acute uveitis without SpA (n=9); and Group 3, SpA without uveitis (n=10). Their fecal calprotectin levels were determined.

Groups 1 and 2 (uveitis patients) were followed by the Ophthalmology Outpatient Clinic of Mackenzie Evangelic Hospital, and G roups 1 and 3 (SpA patients) were followed by the rheumatology unit of the same hospital. The inclusion criterion for uveitis patients was active acute anterior uveitis according to the Standardization of Uveitis Nomenclature (SUN)^(11)^. The inclusion criterion for SpA patients was fulfilling the SpA classification criteria of the International Society for Assessment of SpondyloArthritis (ASAS)^(12,13)^.

Patients with infectious uveitis or those using nonsteroidal anti-inflammatory drugs or oral glucocorticoids at the time of fecal calprotectin specimen collection were excluded. Infectious etiologies were excluded on the basis of a medical history and clinical and laboratory tests.

### Data Collection

Epidemiological and clinical data were obtained through direct questioning and review of charts.

### Measuring Fecal Calprotectin Levels

Fecal calprotectin specimens were collected prior to adopting any new systemic treatment or modifying any current treatment protocols. Specimens from groups 1 and 2 were collected during eye inflammatory activity. The fecal samples were tested using the of Bühlmann (Basel, Switzerland) enzyme-linked immunosorbent assay (ELISA) kit. A short extraction procedure using 50 mg of feces and 2.5 mL of extraction buffer was performed, and then selective measurement of fecal calprotectin by sandwich ELISA was carried out^(14)^. Values below 50 µg/g were not considered indicative of gastrointestinal tract inflammation^(15)^.

### Statistical analysis

The data obtained were collected in tables. To determine data distribution, the Shapiro-Wilk test was performed. The central tendency was expressed as the mean and standard deviation (SD) for parametric data and as the median and interquartile range (IQR) for nonparametric data. Comparison of fecal calprotectin levels among the three groups was done using the Kruskal--Wallis test followed by Tukey’s multiple-comparison test. Comparison of age in the three groups was done using one-way analysis of variance, and assessment of fecal calprotectin levels between users and non-users of tumor necrosis factor alpha (TNF-a) was done using the Mann-Whitney test. Nominal data were compared using Fisher’s and chi-square tests. The adopted significance was 5%. Calculations were performed using GraphPad Prism version 4.0 (GraphPad Software Inc., San Diego, CA, USA).

## RESULTS

The epidemiological, clinical, and treatment data of all groups are given in table 1. Patients in Group 3 were older (p=0.01), human leukocyte antigen b27 (HLA B27) was less common in Group 2 (p=0.0004), and group 2 had a tendency toward more associated intermediate uveitis than other groups (p=0.056).

**Table 1.** Epidemiological, clinical, and treatment data of the studied groups

	SpA with uveitis (n=9)	SpA without uveitis (n=10)	Uveitis without SpA (*) (n=9)	*p*-value
Age (years)				0.01
Range	30–72	32–63	13.0–58.0	
Mean ± SD	45.3 ± 12.8	51.4 ± 9.0	34.0 ± 15.2	
Gender				0.68
Female	6 (66.6%)	5/10 (50%)	6 (66.6%)
Male	3 (33.3%)	5/10 (50%)	3(33.3%)
Spa type				1.00
Psoriatic arthritis	3/9 (33.3%)	4/10 (40%)	-	
Ankylosing spondylitis	6/9 (66.6%)	6/10 (60%)	-	
Hla b27	8/9 (88.8%)	5/7 (71.4%)	0/9	0.0004
Anti TNF alpha users	4/9 (44.4%)	4/10 (40%)	1/9 (11.1%)	0,25
Anterior uveitis	9/9 (100%)		9/9 (100%)	1.00
Associated intermediate uveitis	2/9 (22.2%)	-	7/9 (77.7%)	0.056

*In this group, 8 of 9 patients had idiopathic uveitis, while 1 had sarcoidosis.

SpA, spondyloarthritis; SD, standard deviation.

**Figure 1.**
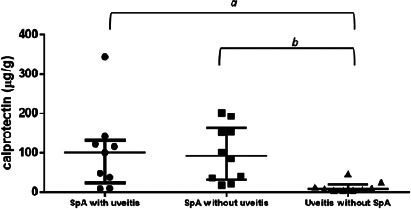
Comparison of fecal calprotectin levels in SpA patients with and without uveitis and in uveitis patients without SpA.

The median fecal calprotectin levels were 101.0µg/g(IQR=24.0−132.5 µg/g) in Group 1, 9.0µg/g(IQR=5−10 µg/g) in Group 2, and 93.0µg/g(IQR=32.2−163.8 µg/g) in Group 1. Both groups of patients with SpA, with or without uveitis, showed higher fecal calprotectin levels than patients with uveitis without SpA (p=0.02). The comparison of fecal calprotectin levels in the three groups is shown in figure 1.

SpA with uveitis: median fecal calprotectin levels = 101.0µg/g(IQR=24.0−132.5 µg/g)

SpA without uveitis: median fecal calprotectin levels = 93.0µg/g(IQR=32.2−163.8 µg/g)

Uveitis without SpA: median fecal calprotectin levels = 9.0µg/g(IQR=5−10 µg/g)

p=0.02 (Kruskal–Wallis test).

*a* and *b* with statistical significance by Tukey’s multiple-comparison test.

SpA, spondyloarthritis; IQR, interquartile range.

## DISCUSSION

Patients with SpA-associated acute uveitis have higher fecal calprotectin levels than those without SpA-associated acute uveitis; these levels are was similar to those found in SpA patients without uveitis. In agreement with our findings, Kang et al. found that 18% of their SpA patients had high fecal calprotectin levels, but there was no relationship between fecal calprotectin levels and the presence of active uveitis or a history of uveitis^(16)^. Biancardi et al. had the same findings while studying uveitis in inflammatory bowel disease^(17)^.

Our findings may lead to two considerations. The first consideration is with regard to concerns the role of intestinal inflammation and the intestinal microbiome in the etiology of anterior uveitis. Rosenbaum et al. studied the role of dysbiosis in uveitis and proposed three hypotheses to explain this interaction:

(a) Abnormal flora may alter intestinal permeability, allowing translocation of microorganisms or their products to the systemic circulation. A bacterial product imprisoned in the iris could activate the immune system and cause inflammation^(10)^.
(b) Bacterial products that enter the systemic circulation could cross-react with ocular tissues due to antigen mimicry, causing immune-mediated reactions^(10)^.
(c) The intestinal microbiome exerts a role in the “education” of the immune system. Gut microorganisms affect the number of T-cells that produce interleukin (IL)-17 and the number of T regulatory cells (Tregs)^(10)^. IL-17 plays a crucial role in the inflammatory process of SpA^(18)^.

Some authors studying animal models of experimental immune-mediated panuveitis have shown that the use of broad-spectrum antibiotics can alter the number of Tregs and decrease the inflammatory eye response^(19)^. This ocular inflammation-gut microbiome interaction is growing fields of inquiry that may help us understand the physiopathology of uveitis and SpA. Our results indicate that high fecal calprotectin levels may be found in SpA-associated uveitis but not in uveitis of other etiologies. Nevertheless, the similar fecal calprotectin levels in SpA with and without uveitis suggest that the underlying SpA is responsible for the high fecal calprotectin levels.

The second consideration is that the use of fecal calprotectin levels may help the clinician classify the etiologies of anterior uveitis. The diagnosis of SpA-associated uveitis is not always easy, as some of the rheumatological findings are incomplete or go unnoticed^(20)^. Uveitis can also be the first manifestation of SpA^(3,18)^. Finding high fecal calprotectin levels may be one more data point to help in this classification. Further studies are necessary to understand the real value of fecal calprotectin levels in the diagnosis of SpA in patients presenting with anterior uveitis.

Although none of our patients had Behçet’s disease, it is necessary to mention that this disease is also associated with intestinal inflammation and elevated fecal calprotectin levels^(21)^. Further studies comparing the fecal calprotectin levels in uveitis patients with SpA and Behçet’s disease may elucidate the role of fecal calprotectin levels in the differential diagnosis of these two diseases.

The present study had several limitations. This was a cross-sectional study. It was difficult to analyze whether having uveitis may cause a further increase in fecal calprotectin levels; our sample was quite small and was not able to demonstrate any differences. Therefore, prospective studies with more patients and serial measurements of fecal calprotectin levels according to the evolution of uveitis are required. In addition, we did not perform gastrointestinal endoscopy on patients with high fecal calprotectin levels; this would have helped us understand the link between the degree of intestinal mucosal inflammation and fecal calprotectin levels. However, it did bring into question the role of fecal calprotectin levels as a possible test to be performed in cases of uveitis of unknown origin. Further studies would be helpful to better clarify this relationship.

Investigation of our series of uveitis patients has shown that those with associated SpA have high fecal calprotectin levels, while those with uveitis without asso ciated SpA show no measurable intestinal alterations. The increase in fecal calprotectin levels in patients with SpA occur regardless of the presence of anterior uveitis.
